# Projected impacts of climate change on snow leopard habitat in Qinghai Province, China

**DOI:** 10.1002/ece3.8358

**Published:** 2021-11-18

**Authors:** Jia Li, Yadong Xue, Charlotte E. Hacker, Yu Zhang, Ye Li, Wei Cong, Lixiao Jin, Gang Li, Bo Wu, Diqiang Li, Yuguang Zhang

**Affiliations:** ^1^ Institute of Desertification Studies Chinese Academy of Forestry Beijing China; ^2^ Research Institute of Forest Ecology, Environment and Protection Chinese Academy of Forestry Beijing China; ^3^ Key Laboratory of Biodiversity Conservation of National Forestry and Grassland Administration Beijing China; ^4^ Department of Biological Sciences Duquesne University Pittsburgh Pennsylvania USA; ^5^ Research Institute of Nature Protected Areas Chinese Academy of Forestry Beijing China; ^6^ Social Information Department of CCTV News Center China Media Group Beijing China

**Keywords:** adaptive strategies, habitat corridors, National park, suitable habitat, vulnerability

## Abstract

Assessing species’ vulnerability to climate change is a prerequisite for developing effective strategies to reduce emerging climate‐related threats. We used the maximum entropy algorithm (MaxEnt model) to assess potential changes in suitable snow leopard (*Panthera uncia*) habitat in Qinghai Province, China, under a mild climate change scenario. Our results showed that the area of suitable snow leopard habitat in Qinghai Province was 302,821 km^2^ under current conditions and 228,997 km^2^ under the 2050s climatic scenario, with a mean upward shift in elevation of 90 m. At present, nature reserves protect 38.78% of currently suitable habitat and will protect 42.56% of future suitable habitat. Current areas of climate refugia amounted to 212,341 km^2^ and are mainly distributed in the Sanjiangyuan region, Qilian mountains, and surrounding areas. Our results provide valuable information for formulating strategies to meet future conservation challenges brought on by climate stress. We suggest that conservation efforts in Qinghai Province should focus on protecting areas of climate refugia and on maintaining or building corridors when planning for future species management.

## Graphical abstract

Climate change challenges current snow leopard conservation efforts; identify refugia and corridors under climate change for species; and provide adaptation strategies to mitigate climate‐related threats to the species.

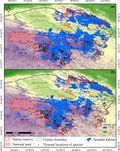

## 1 INTRODUCTION

Earth's climate is facing unprecedented changes (Garcia et al., 2014). Recent assessments by the Intergovernmental Panel on Climate Change (IPCC) have reported that the global average temperature has increased by 0.85 ᵒC over the last one hundred years and that warming trends will continue in the near future regardless of attempts to curtail greenhouse gas emissions (IPCC, 2014). This has emerged as a key threat for global biodiversity conservation (Arneth et al., 2020). Previous studies have shown the impacts of climate change on wildlife population dynamics (Martay et al., 2017; Tanentzap et al., 2020), distribution (Pastor, 2016; Sántiz et al., 2016), phenology (Koleček et al., 2020), evolution (Karell et al., 2011 ), and invasive species (Bertelsmeier et al., 2013). These impacts are expected to be exacerbated in future (Bellard et al., 2012). Natural selection provides a mechanism whereby species can adapt to changes in their environments (Rinawati et al., 2013). Species persistence depends on the ability to reduce or avert climate change challenges through dispersal to suitable climatic areas, or through ecological plasticity (e.g., morphological, physiological, or behavioral traits) and evolutionary responses to global changes (Bellard et al., 2012; Charmantier et al., 2008; Foden et al., 2016; Seebacher et al., 2015). However, because of existing stressors, many species have reduced capacity to cope and will be at an increased risk of extinction (Schloss et al., 2012). One synthesis estimated that one in six species are likely to be at increasingly high risk of extinction if a “business as usual” mentality surrounding carbon emissions continues (Urban, 2015).

It is imperative to identify the species and habitats that are most vulnerable to climate change (Foden et al., 2013). This poses an immense challenge for conservation biologists. Accurately predicting the impacts of climate change is difficult (Foden et al., 2018). Assessments of species vulnerability based on species distribution models (SDMs, such as MaxEnt) can be valuable tools for management and conservation (Li et al., 2018; Rowland et al., 2011). These modeling approaches correlate current species range and variables retrieved from databases to forecast shifts in species’ distribution under future climate scenarios (Guisan et al., 2013; Zhang, Clauzel, et al., 2019). The vulnerability of a species can then be assessed by comparing the differences in currently suitable versus future suitable habitat areas (Arribas et al., 2012; Sudo et al., 2019). Forecasts of large habitat contractions, expansions, or minimal overlap between current and future ranges can be used as indexes of species vulnerability (Carvalho et al., 2010; Guisan & Thuiller, 2005). Several analyses have provided examples of species likely to be vulnerable to climate change. For example, Hatten et al. (2016) used SDMs to identify bird and reptile vulnerabilities in the southwestern United Sates and found that nearly two‐thirds of the species’ suitable habitat was predicted to contract. Li et al. (2017) used modeling to assess the vulnerability of giant pandas (*Ailuropoda melanoleuca*) to future climate change and subsequently identified potential refugia and habitat corridors to be used in future conservation action plans.

The snow leopard (*Panthera uncia*) occupies high mountain regions in Central Asia. Their distribution covers an area of more than 1.2 million km^2^ across 12 countries (Snow Leopard Network, 2014). The wild population consists of approximately 4000–7000 individuals (Li et al., 2016; Snow Leopard Network, 2014), although there is substantial uncertainty (Ale & Mishra, 2018; Liu et al., 2019). The snow leopard was listed as Endangered on the IUCN red list from 1972 to 2017 and is currently listed as Vulnerable (McCarthy et al., 2017). The primary threats to snow leopard survival include prey depletion, poaching and retaliatory killing, and habitat loss and fragmented due to climate change and human settlement expansion (Aryal, 2017; Li et al., 2016; McCarthy et al., 2017; Valentová, 2017). Although conservation efforts have improved snow leopard populations in some areas (Liu et al., 2016; Xu et al., 2008), the metapopulation continues to show a decreasing trend in number of individuals. To date, studies of snow leopard ecology have included population genetics (Janecha et al., 2014; Karamachary et al., 2011; Zhang, Hacker, et al., 2019; Zhou et al., 2014), habitat selection (Weiskopf et al., 2016; Xu et al., 2006), foraging strategy (Anwar et al., 2011; Hacker et al., 2021; Suryawanshi et al., 2017), activity (McCarthy et al., 2005; Tang et al., 2017), and home range (Johansson et al., 2016). In recent years, the snow leopard has attracted public attention and studies on its conservation have diversified, now including climate change (Forrest et al., 2012; Li et al., 2016), human‐snow leopard conflict (Hacker et al., 2020; Li et al., 2013; Sullivan et al., 2018), illegal trade (Ma, 2012; Maheshwari & Niraj, 2018), and conservation landscapes (Li et al., 2020). However, data remain limited in comparison to other charismatic species (Suryawanshi et al., 2019).

China holds over half of currently known suitable snow leopard habitat and plays a vital role in maintaining the entirety of the snow leopard population (McCarthy & Chapron, 2003). The snow leopard population size and distribution suffered extensively due to poaching and illegal trade from the 1950s to 1980s in China (Li et al., 2013; Valentová, 2017). Over the past few decades, the Chinese government has implemented conservation action plans to protect wildlife, including snow leopards. This has involved the strengthening of laws against poaching and trade, the establishment of nature reserves and pastureland rehabilitation (Li & Lü, 2014; Li et al., 2013). At present, most pre‐existing key threats and limiting factors for species across China are being mitigated. For snow leopards, populations are even recovering in some areas (e.g., the Sanjiangyuan region; Liu et al., 2016). However, most areas in snow leopard habitat (e.g., the Qinghai‐Tibetan Plateau) are ecosystems vulnerable to climate change (Peng et al., 2012; Yuke, 2019), and the World Wildlife Fund for Nature (WWF) has stated that more than a third of current snow leopard habitat may be lost due to increasing global warming (Sharma et al., 2015). Previous work surrounding climate change impacts on the snow leopard have been done in China. Li et al. (2016) found that 26% of currently suitable habitat in China would be lost under a mild climate scenario. However, the occurrence data used in the study only went to the year 2008, and thus, more recent assessments are needed. Forrest et al. (2012) investigated climate change in the Himalayas and found that 25% of snow leopard habitat in southern China would be lost under a high emissions scenario. While this area is one of the most important areas in China for snow leopard distribution, it represents only a small portion of known habitat. Other areas of China, such as Qinghai Province, also require fine scale investigation in regard to climate change.

Qinghai Province holds some of the largest suitable stretches of continuous snow leopard habitat and is a very important distribution area in China (Liu et al., 2016). In this province, a total of 28 nature reserves have been established to protect snow leopards and other sympatric species (Zheng, 2011). In addition, the Sanjiangyuan and Qilian mountain reserves were upgraded as pilot national parks in 2017, integrating current nature reserves to resolve the problems of fragmented distribution and management. Given this area's importance to the species in China, it is critical to assess the vulnerability of the snow leopard to future climate change in Qinghai Province and to adopt adaptive conservation strategies to reduce its adverse impacts. Here, we built a snow leopard distribution model based on current species occurrence data and associated bioclimatic and environmental variables. We aimed to (a) identify currently suitable snow leopard habitat and distribution areas, and assess the vulnerability of snow leopard habitat in Qinghai Province to climate change from the current period to the 2050s; (b) identify climate refugia that could support the population during periods of climatic stress; (c) establish habitat corridors that could facilitate individual movement among patches of suitable habitat; and (d) determine how protected areas will be impacted by climate change, and provide conservation strategies under the future climate change scenario.

## 2 METHODS

### 2.1 Study area

**FIGURE 1:**
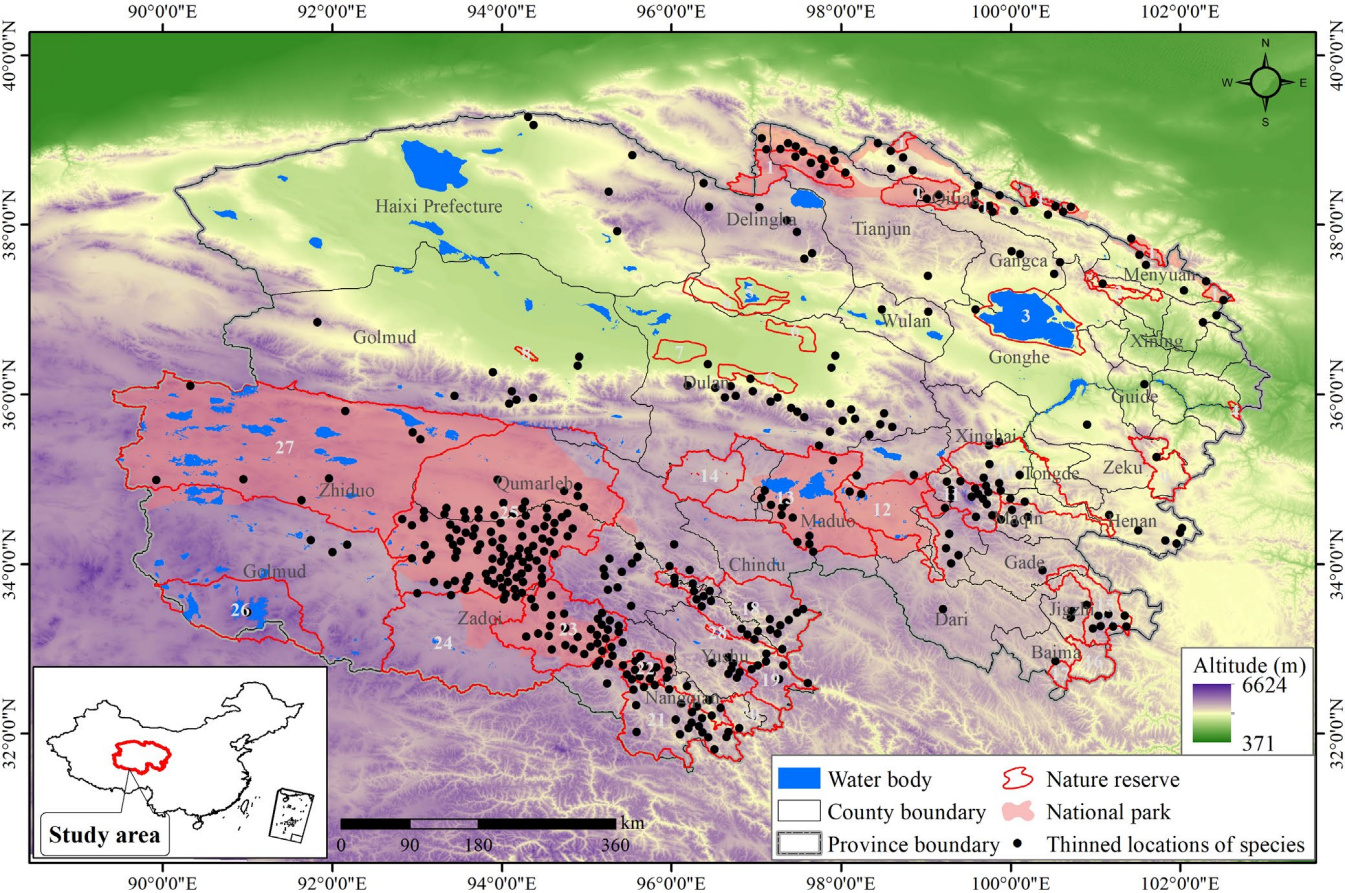
Mapping of the study area in China, thinned localities of snow leopard, nature reserves, and national parks in Qinghai Province. Codes of the reserves: 1‐Qilian Mountain; 2‐Datongbeichuanheyuan; 3‐Qinghai lake; 4‐Mengda; 5‐Keluke lake; 6‐Suosuolin; 7‐Nuomuhong; 8‐Huyang forest; 9‐Sanjiangyuan‐Maixiu; 10‐Sanjiangyuan‐Zhongtie Jungong; 11‐Sanjiangyuan‐Animaqin; 12‐Sanjiangyuan‐Xingxing lake; 13‐Sanjiangyuan‐Zalinghu Elinghu lake; 14‐Sanjiangyuan‐Yueguzonglie; 15‐Sanjiangyuan‐Nianbao Yuze; 16‐Sanjiangyuan‐Make river; 17‐Sanjiangyuan‐Duoke river; 18‐Sanjiangyuan‐Tongtian river; 19‐Sanjiangyuan‐Dongzhong; 20‐Sanjiangyuan‐Jiangxi; 21‐Sanjiangyuan‐Baiza; 22‐Sanjiangyuan‐Ansai; 23‐Sanjiangyuan‐Guozong Muzha; 24‐Sanjiangyuan‐Dangqu; 25‐Sanjiangyuan‐Suogartoma river; 26‐Sanjiangyuan‐Gela Dangdong; 27‐Hoh Xil; 28‐Longbao

Qinghai Province is located in the northeastern corner of the Qinghai–Tibetan Plateau in China (Figure 1). It covers an area of 720,000 km^2^, with a population of 5 million people (Zheng, 2011). Qinghai Province holds the source of the Yellow River and Yangtze River, as well as the upper reach of the Lancang (Mekong) River. The area consists primarily of grasslands, rangelands, deserts, and wetlands, with most mountains having an average altitude of above 4000 m. The climate is a typical plateau continental climate, dry and cold and with long winters, short summers, frequent winds, little rainfall, long hours of sunshine, and stark differences in temperature between day and night. The average annual temperature is −4°C (ranging from −20°C in January to 8°C in July), the average annual rainfall is 150–420 mm, and a clear warming trend with increased precipitation has occurred over the past several decades (Peng et al., 2012). The primary mammal species in addition to the snow leopard in Qinghai Province include the Eurasian lynx (*Lynx lynx*), Tibetan brown bear (*Ursus arctos*), desert cat (*Felis bieti*), wild yak (*Bos mutus*), Tibetan antelope (*Pantholops hodgsonii*), blue sheep (*Pseudois nayaur*), alpine musk deer (*Moschus chrysogaster sifanicus*), and the Eurasian otter (*Lutra lutra*; Zheng, 2011).

### 2.2 Species records

A total of 1291 snow leopard occurrence records in Qinghai Province were obtained from camera‐trap monitoring, signs (including footprints, scrapes), genetically verified scat samples, and published literature from 2005 to 2020 (see details in Appendix S1; Figure 1). Because these occurrence records are from multiple unplanned surveys, the records likely display spatial autocorrelation (Kramer‐Schadt et al., 2013). To correct for this, we thinned occurrence records using a thinning distance of 10 km (Radosavljevic & Anderson, 2013). To correct for the effect of sample selection bias on predictive performance (Vanderwal et al., 2009), we randomly generated 10,000 pseudo‐absence points with the exclusion of a 40 km (the maximum daily movement distance of a snow leopard; McCarthy et al., 2005) buffer area from snow leopard occurrence records in the study area to consider dispersal ability in both suitable habitat and gap analyses.

### 2.3 Environmental variables

Nineteen bioclimatic variables for current (average for 1950–2000) and future (average for 2041–2060, henceforth referred to as the 2050s) climate conditions were downloaded from the WorldClim database (available at http://www.worldclim.org) at 30‐second resolution. The future projection was extracted from HadGEM_2_‐AO general circulation models of the IPCC‐CMIP_5_ under the representative concentration pathway (RCP4.5; Beak et al., 2013). RCP4.5 represents a mild climate change scenario with increases in the average global temperature of 0.9–2.0°C for the 2050s, which would fall within a 2°C global warming limit (UNFCCC, 2015). The time horizon of the 2050s was selected as this period is far enough into the future for marked changes to have occurred (Young et al., 2012).

Additional environmental variables at the same resolution were used to construct snow leopard distribution models. The density of settlements, roads, railways, and rivers were obtained from the Resource and Environment Science and Data Center of China website (http://www.resdc.cn/Default.aspx). Elevation was obtained from a digital elevation model with 30‐second resolution from the WorldClim database, and slope data derived from elevation (see details in Appendix 1). Nonclimatic variables were unavailable for the 2050s, and thus, we kept these variables static in our projections (Li et al., 2017; Stanton et al., 2012).

All variables were resampled at a 1‐km^2^ resolution and put into the same projection using ArcGIS 10.4 (ESRI Inc.). SDMtoolbox (Brown, 2014) was used to determine multicollinearity of variables. One variable of each relational pair was removed where Pearson's correlation coefficients of |*r*| > .6 (Appendix 2; Cord et al., 2014). The most biologically meaningful variables to the snow leopard were retained (Bai et al., 2018; Li et al., 2016).

### 2.4 Habitat suitability model

Maximum Entropy Models (MaxEnt software version 3.3.3k) are considered a useful and superior tool to build habitat suitability models with presence‐only data (Elith et al., 2011). With this model, performance is measured under the receiver operating characteristic curve (AUC) with a value closer to 1 representing a near perfect model. Snow leopard occurrence data were divided into random training tests (AUC_test_: 75%) and testing tests (AUC_train_: 25%). We ran 10 replicates and conducted a subsample procedure with recommended default settings. Variable importance was estimated by the permutation importance method (Wisz et al., 2008). The predicted area under the curve (AUC) was used as the performance estimator of the model. The logistic results of the MaxEnt model were considered to represent the probabilities of species occurrence (Phillips et al., 2006). We then binarized potential presence/absence areas according to the maximum training sensitivity average plus the specificity logistic threshold (Li et al., 2018; Songer et al., 2012). Areas with probability values above the threshold were regarded as suitable snow leopard habitat. Based on criteria of prioritized protecting habitat set by Global Snow Leopard and Ecosystem Protection Program (Snow Leopard working Li et al., 2020; Secretariat, 2013), we defined continuous area larger than 10,000 km^2^ as core suitable habitat (calculated in ArcGIS 10.4).

### 2.5 Gap analysis of protected areas

Networks of protected areas remain the most valuable resource for snow leopard persistence. We overlapped current and future suitable habitats with the borders of established nature reserve and national parks to explore the conservation effectiveness of these reserves in protecting snow leopards under the future climate change scenario using ArcGIS 10.4.

### 2.6 Vulnerability assessment

We assessed changes in suitable habitat between the current time frame and the 2050s and categorized resulting habitat as follows: (i) Unsuitable habitat: area where habitat that was not suitable for snow leopards in current and future climate projections; (ii) Vulnerable habitat: area in which currently suitable habitat was projected to be unsuitable in the 2050s; (iii) Climate refugia: area where currently suitable habitat overlapped with predicted suitable habitat for the 2050s; (iv) Newly suitable habitat: area where currently unsuitable habitat was predicted to become suitable by the 2050s.

Three indicators were used to assess habitat vulnerability: (i) percentage of suitable habitat area change (AC); (ii) percentage of currently suitable habitat area lost by the 2050s (SH_L_); and percentage of increased suitable habitat area for the 2050s (SH_I_). Indicators were calculated as follows:

$$\text{AC}=\left({\text{SH}}_{f}{\;\text{- SH}}_{c}\right){\text{/SH}}_{c}\times 100\%$$

$${\text{SH}}_{L}=\left({\text{SH}}_{c}{\;\text{- SH}}_{o}\right){\text{/SH}}_{c}\times 100\%$$

$${\text{SH}}_{I}=\left({\text{SH}}_{f}{\;\text{- SH}}_{o}\right){\text{/SH}}_{f}\times 100\%$$

For the formulas above, SH_f_ is the area of predicted suitable habitat for snow leopard under the 2050s climatic scenario; SH_c_ is the area of currently suitable habitat; and SH_o_ is the area of overlapped suitable habitat between present time and the 2050s (Li et al., 2018; Thuiller et al., 2005).

We used a Mann–Whitney *U* test to compare the change in average suitable habitat elevation between the current time frame and the 2050s. Data were expressed as mean ± standard error (Mean ± SE). Statistical significance was considered at *p *< .05. Statistical analyses were performed in SPSS 19.0 (IBM Inc.).

### 2.7 Habitat connectivity analysis

Linkage Mapper identifies wildlife corridors and suitable habitat patches via connectivity analyses (McRae & Kavanagh, 2011). The inverse of the logistic output from the resulting MaxEnt model was used as a measure of movement resistance for the snow leopard and least‐cost linkages were mapped between core habitat areas (>500 km^2^ based on the mean home range of snow leopard; Johansson et al., 2020). The least‐cost pathways (LCP) and corridors were implemented in the Linkage Mapper toolbox (ArcGIS 10.0‐10.6; https://circuitscape.org/linkagemapper/).

## 3 RESULTS

### 3.1 Species distribution model

A total of 400 occurrence records and eight variables were used in the final MaxEnt model as model parameters. The habitat suitability model provided satisfactory results with an average training AUC value of 0.906 (±0.004) and average testing AUC of 0.895 (±0.016). The percent contribution of each model variable ranked from highest to lowest was as follows: annual mean temperature (23.8%), density of rivers (22.3%), temperature constancy (16.9%), precipitation of driest month (12.7%), mean diurnal range (12.7%), density of settlements (10.5%), slope (2.9%), and density of railways (1.5%). The average threshold value for the measure of suitable habitat was 0.336.

### 3.2 Changes in habitat suitability

**FIGURE 2:**
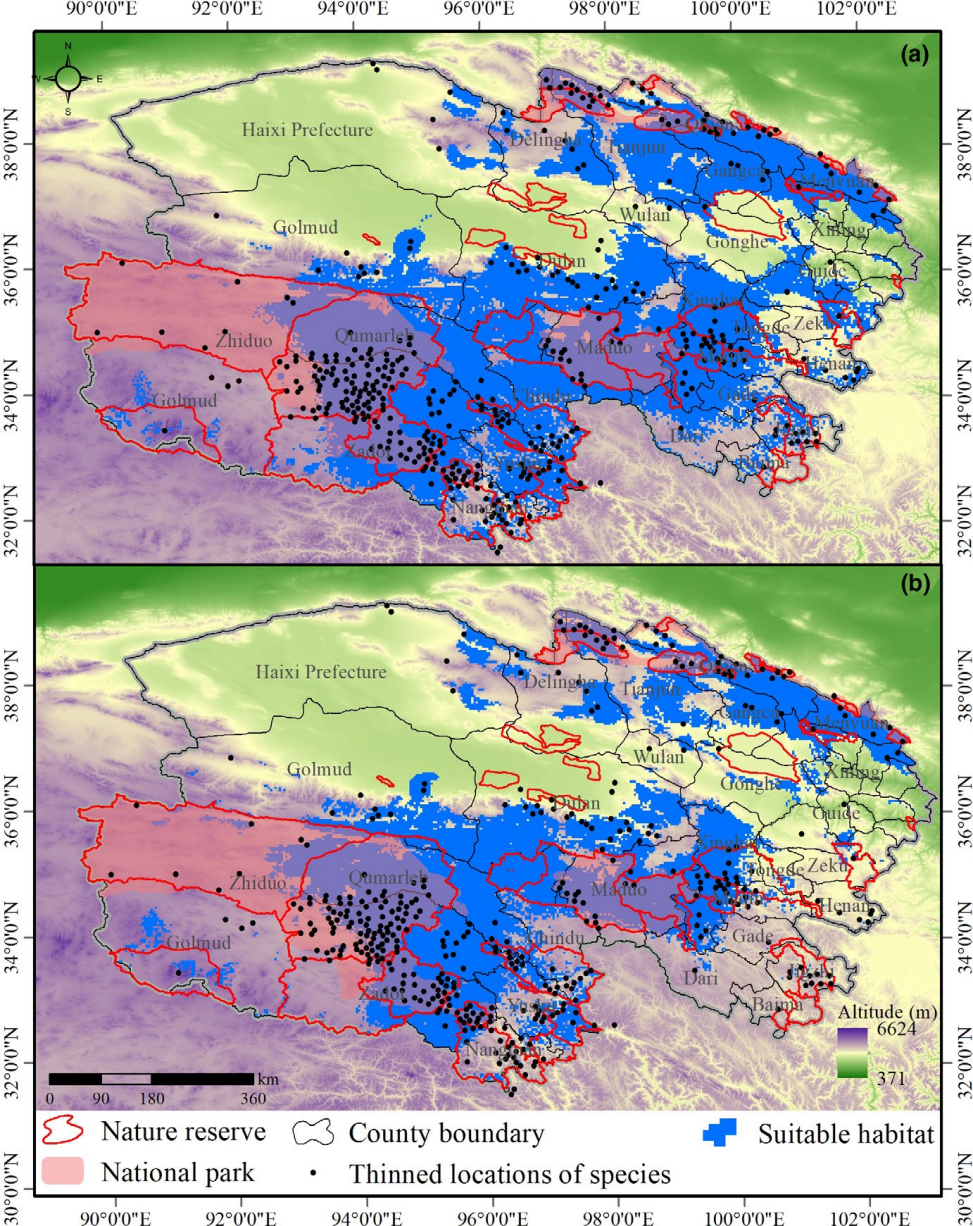
Suitable snow leopard habitat distributed in the nature reserves and national parks of Qinghai Province. (a) Suitable habitat under the current climate scenario; (b) predicted habitat suitability by the 2050s under a future mild climate change scenario

Under current conditions, the area of suitable snow leopard habitat in Qinghai Province was 302,821 km^2^ (Table 1; Figure 2), predominately distributed in 31 counties (area of suitable habitat larger than 1000 km^2^). In Tianjun, Qilian, Xinghai, Dulan, Zadoi, Chindu, Zhiduo, Qumarleb, Maqin, Maduo, Yushu, Zhiduo, and Golmud, the area of suitable habitat was larger than 10,000 km^2^ and appears to be distributed in continuous patches. By the 2050s, the area of suitable habitat decreased by 24.38% (Area Change, AC) to 228,997 km^2^. Suitable habitat areas larger than 1000 km^2^ dropped from 31 to 22 counties, and only 9 had more than 10,000 km^2^ (Tianjun, Qilian, Dulan, Zadoi, Zhiduo, Qumarleb, Maduo, Golmud, and Dari).

**TABLE 1:** Predictions of the suitable habitat area (km^2^) for snow leopard in Qinghai Province in the current period and the 2050s

City or prefecture	County	Suitable habitat area		Percentage of area change (AC)
		Current	2050s	
Xining city	Datong	1916.17	1392.50	−27.33%
	Huanzhong	110.15	107.80	−2.14%
	Huanyuan	337.97	601.41	77.95%
Haidong city	Ledo	665.49	433.60	−34.84%
	Minhe	92.51	17.78	−80.78%
	Huzhu	1916.24	1703.03	−11.13%
	Hulong	273.40	97.82	−64.22%
	Xunhua	165.62	19.65	−88.14%
Haibei Tibetan Autonomous Prefecture	Menyuan	5532.31	5532.31	0.00%
	Qilian	10,351.10	10,347.40	−0.04%
	Haiyan	2362.68	1831.53	−22.48%
	Gangca	9345.95	6039.00	−35.38%
Hainan Tibetan Autonomous Prefecture	Tongren	1838.93	211.31	−88.51%
	Jianza	374.51	0.00	−100.00%
	Zeku	1867.85	368.66	−80.26%
	Henan	1740.57	34.36	−98.03%
	Gonghe	3835.11	2233.76	−41.75%
	Tongde	2124.52	631.43	−70.28%
	Guide	1218.24	405.06	−66.75%
	Xinghai	10,507.46	8425.29	−19.82%
	Guinan	4839.81	308.27	−93.63%
Guoluo Tibetan Autonomous Prefecture	Maqin	12,059.51	8319.80	−31.01%
	Baima	1279.68	0.00	−100.00%
	Gade	5318.43	464.17	−91.27%
	Dari	6382.37	1111.95	−82.58%
	Jigzhi	3374.80	153.14	−95.46%
	Maduo	23,856.65	19,789.76	−17.05%
Yushu Tibetan Autonomous Prefecture	Yushu	12,165.75	8154.52	−32.97%
	Zadoi	25,520.86	20,953.95	−17.89%
	Chindu	12,501.97	5147.07	−58.83%
	Zhiduo	29,266.04	28,247.93	−3.48%
	Nangqian	4360.34	1859.18	−57.36%
	Qumarleb	35,788.15	35,553.55	−0.66%
Haixi Mongolian and Tibetan Autonomous Prefecture	Haixi	3562.89	3651.17	2.48%
	Golmud	15,126.80	16,463.61	8.84%
	Delingha	7136.88	5544.70	−22.31%
	Wulan	606.62	278.79	−54.04%
	Dulan	25,546.31	20,204.27	−20.91%
	Tianjun	17,551.16	12,358.01	−29.59%

Note

Under the current conditions, area of suitable habitat for snow leopard in Qinghai Province was 302,821 km^2^, and dramatically reduced to 228,997 km^2^ by the 2050s due to climate change, representing a decreased of 24.38%.

**FIGURE 3:**
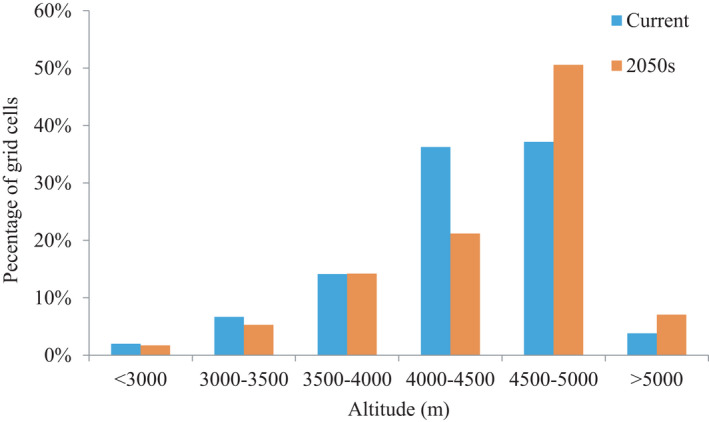
Impacts of climate change on the potential distribution of snow leopard in Qinghai Province for elevation. The *y*‐axis represents the percentage of grid cells with a 500 m elevation interval

Climate change would also result in an upward shift of suitable habitat (Figure 3). The average elevation of suitable habitat in the 2050s was projected to be 4395 ± 478 m, which is significantly higher (*z *= −17.91, *p* < .001) than that of current suitable habitat (4306 ± 503 m).

### 3.3 Coverage of protected areas

Results showed that current nature reserves protected 38.78% of currently suitable habitat and 42.56% suitable habitat in the 2050s (Table 2; Figure 2). By the 2050s, 23 nature reserves will have a decrease in suitable snow leopard habitat, among which Maixiu (AC = −77.97%), Zhongtie Jungong (AC = 43.22%), Nianbao Yuze (AC = 94.68%), Tongtian river (AC = −44.09%), Dongzhong (AC = −15.63%), Jiangxi (AC = 50.35%), Baiza (AC = −53.62%), Dangqu (AC = −61.78%), Suogartoma river (AC = −7.58%), and Gela Dangdong (AC = −23.94%) nature reserves are estimated to suffer most extensively. Climate change will only scarcely increase the distribution of snow leopards in some nature reserves, mainly in Hoh Xil (AC = 29.06%), Ansai (AC = 19.78%), and Yueguzonglie (AC = 3.97%).

**TABLE 2:** The predicted changes in suitable habitat for snow leopard in nature reserves and national parks

Nature reserve	Suitable habitat area		Percentage of area change (AC)
	Current	2050s	
1‐Qilian mountain	4857.62	4834.75	−0.47%
2‐Datongbeichuanheyuan	1082.03	816.73	−24.52%
3‐Qinghai lake	289.36	0.00	−100.00%
4‐Mengda	0.00	0.00	–
5‐Keluke lake	0.00	0.00	–
6‐Suosuolin	47.51	0.00	−100.00%
7‐Nuomuhong	0.00	0.00	–
8‐Huyang forest	0.00	0.00	–
9‐Sanjiangyuan‐Maixiu	1478.10	325.63	−77.97%
10‐Sanjiangyuan‐Zhongtie Jungong	6954.34	3948.92	−43.22%
11‐Sanjiangyuan‐Animaqin	4265.19	4127.19	−3.24%
12‐Sanjiangyuan‐Xingxing lake	6782.51	6777.70	−0.07%
13‐Sanjiangyuan‐Zalinghu Elinghu lake	13,525.77	12,545.41	−7.25%
14‐Sanjiangyuan‐Yueguzonglie	3894.50	4049.18	3.97%
15‐Sanjiangyuan‐Nianbao Yuze	1757.59	93.54	−94.68%
16‐Sanjiangyuan‐Make river	80.06	0.00	−100.00%
17‐Sanjiangyuan‐Duoke river	85.82	0.00	−100.00%
18‐Sanjiangyuan‐Tongtian river	8172.06	4568.65	−44.09%
19‐Sanjiangyuan‐Dongzhong	2672.43	2254.70	−15.63%
20‐Sanjiangyuan‐Jiangxi	1103.84	548.01	−50.35%
21‐Sanjiangyuan‐Baiza	2853.42	1323.32	−53.62%
22‐Sanjiangyuan‐Ansai	1228.43	1471.47	19.78%
23‐Sanjiangyuan‐Guozong Muzha	10,020.99	9593.62	−4.26%
24‐Sanjiangyuan‐Dangqu	7809.32	2984.43	−61.78%
25‐Sanjiangyuan‐Suogartoma river	32,328.48	29,879.40	−7.58%
26‐Sanjiangyuan‐Gela Dangdong	1027.30	781.31	−23.94%
27‐Hoh Xil	5050.83	6518.50	29.06%
28‐Longbao	82.28	27.54	−66.53%
Sanjiangyuan National Park	66,294.78	61,259.74	−7.59%
Qilian Mountain National Park	9518.84	9513.21	−0.06%

Note

Current nature reserves protected 38.78% of currently suitable habitat and 42.56% of suitable habitat in the 2050s.

With regard to national parks, Sanjiangyuan and Qilian Mountain protected 25% of currently suitable habitat area and 30.9% of suitable habitat area in the 2050s (Table 2; Figure 2). Sanjiangyuan national park will lose 7.59% (AC) of its suitable habitat by the 2050s, but suitable habitat in Qilian Mountain will remain stable and is an area of climate refugia.

### 3.4 Vulnerability assessment

**FIGURE 4:**
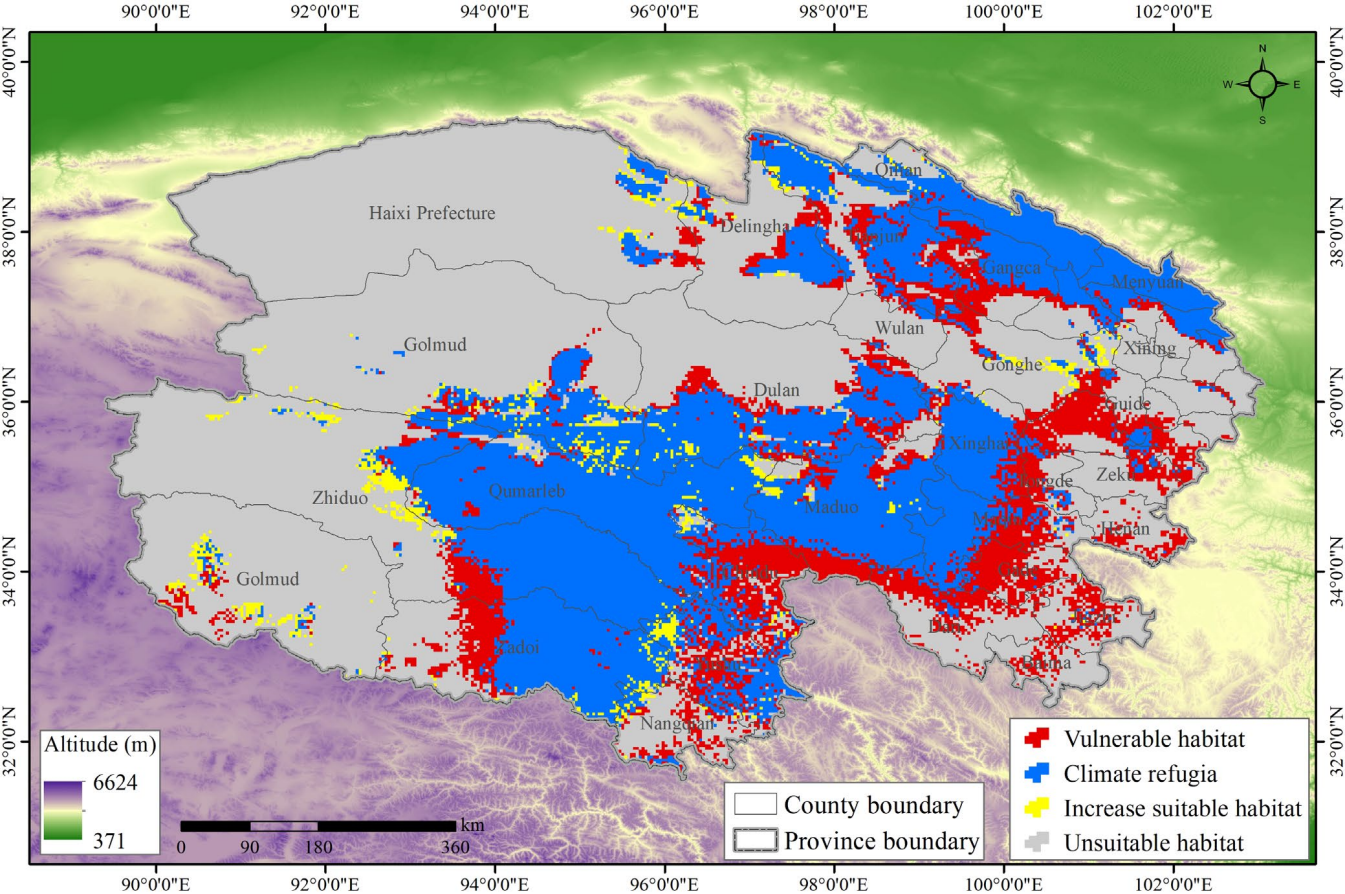
Vulnerability levels of suitable habitat for snow leopard. A total 30% of currently suitable habitat for snow leopard would be vulnerable to climate change in Qinghai Province

Our model predicted that 90,480 km^2^ (SH_L_ = 30%) of currently suitable habitat for the snow leopard is vulnerable to climate change (Figure 4). Unchanged suitable habitat (i.e., climate refugia) covers an area of 212,341 km^2^ and is mainly distributed in the counties of Qumarleb, Maduo, Zadoi, Yushu, Xinhai, Dulan, and Golmud in Sanjiangyuan, and Menyuan, Gangca, and Tianjun in Qilian Mountain. Our results also showed an increase in suitable habitat (16,656 km^2^; SH_I_ = 7.27%) scattered throughout Zhiduo, Golmud, and Gonghe.

### 3.5 Gap analysis

**FIGURE 5:**
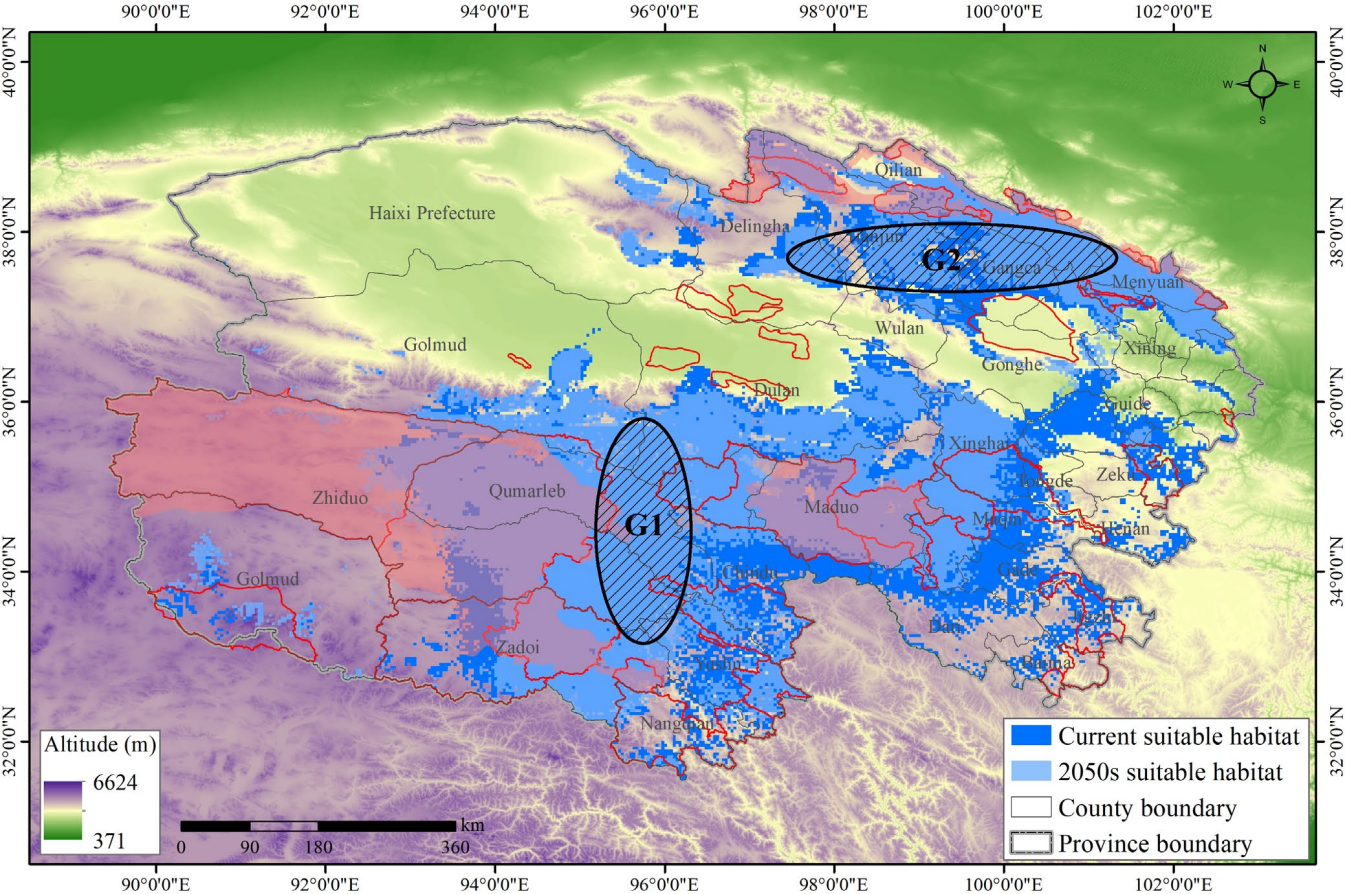
Protection gaps within the current conservation network for suitable snow leopard habitat in Qinghai Province. G1–G2 indicates protection gaps

Gap analysis showed areas of suitable habitat at the junction of Qumarlep and Chindu and surrounding areas (G1; Figure 5), and at the junction of Qilian, Gangca and Tianjun (G2; Figure 5). These areas are largely unprotected, leaving significant gaps in the current conservation network. Reserves should be established in these locations to provide suitable habitat patches that are stable and can contribute to the improvement of habitat connectivity.

### 3.6 Potential habitat corridors

**FIGURE 6:**
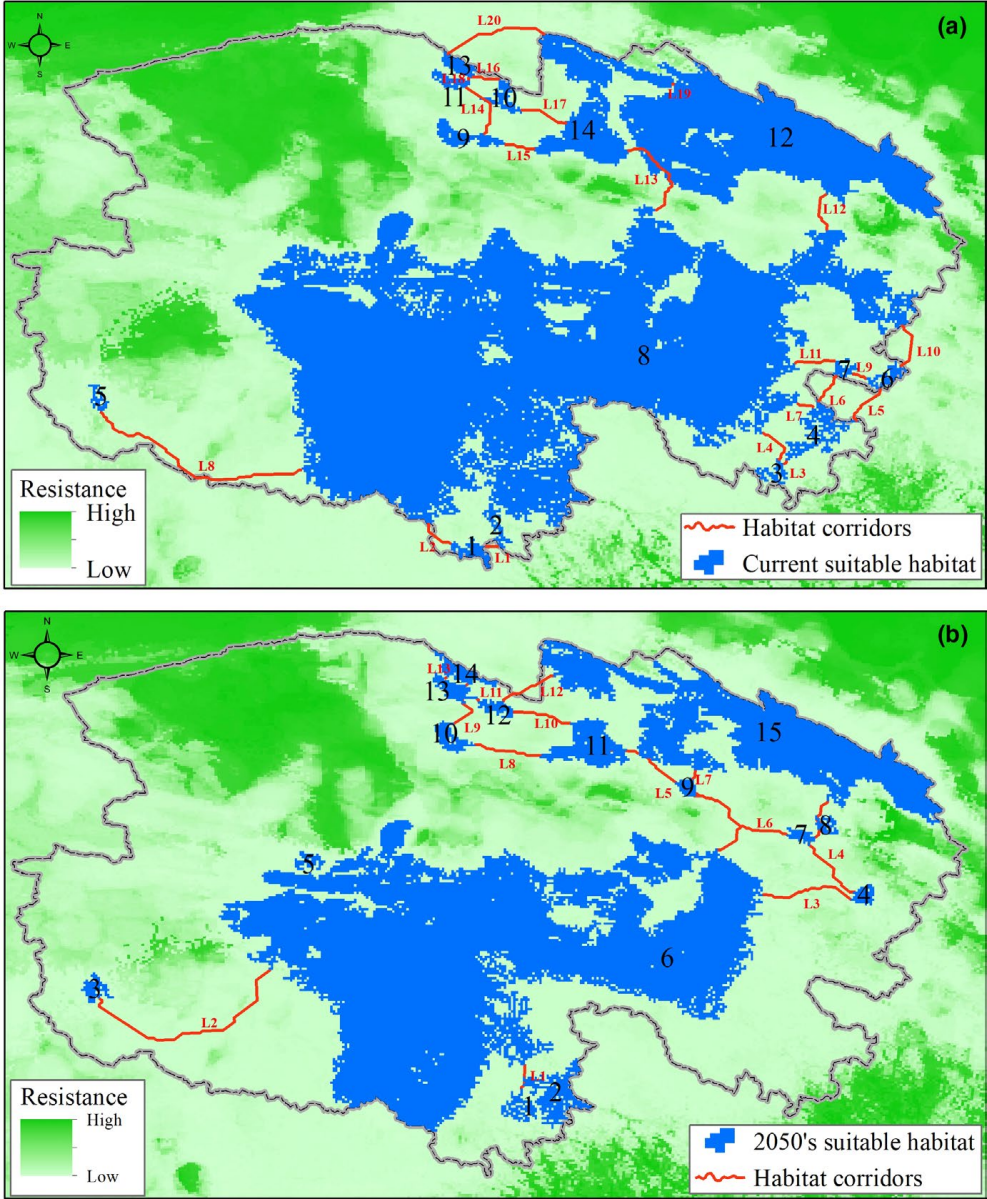
The potential corridors between current and future core suitable habitat patches for snow leopard in Qinghai Province

Suitable habitat patches for the snow leopard connected by least‐cost pathways (LCP) represent potential corridors between core habitat areas (>500 km^2^). Linkage Mapper mapped 20 least‐cost potential corridors between currently suitable habitat patches (Table 3; Figure 6), with the average LCP being 68 km (ranging from 4 to 322 km). Linkage Mapper mapped 13 least‐cost potential corridors in the 2050s, with the average LCP being 127 km (ranging from 5 to 444 km).

**TABLE 3:** Characteristic of linkages between snow leopard suitable habitat patches in Qinghai Province in the current period and the 2050s

Currently suitable habitat patches				2050s suitable habitat patches			
HC ID	From SHP	To SHP	LCP (km)	HC ID	From SHP	To SHP	LCP (km)
L1	1	2	18	L1	1	6	44
L2	1	8	52	L2	3	6	326
L3	3	4	9	L3	4	6	126
L4	3	8	63	L4	4	15	196
L5	4	6	74	L5	6	10	444
L6	4	7	44	L6	7	9	149
L7	4	8	24	L7	9	15	23
L8	5	8	322	L8	10	11	86
L9	6	7	21	L9	10	14	87
L10	6	8	82	L10	11	12	75
L11	7	8	53	L11	12	13	9
L12	8	12	72	L12	12	15	78
L13	8	14	148	L13	13	14	5
L14	9	11	97				
L15	9	14	39				
L16	10	13	34				
L17	10	14	62				
L18	11	13	7				
L19	12	14	4				
L20	13	14	134				

Abbreviations: HC, Habitat corridor; LCP, Least cost patch length; SHP, Suitable habitat patch.

The establishment of potential corridors will be a long‐term process and does not address the more urgent needs for species conservation. Rather, habitat corridors should be based on increasing the likelihood for known isolated populations to shift into core suitable habitat patches (L14–L18 and L3–L7; Figure 6) and assisting genetic exchange between the Sanjiangyuan and Qilian Mountain populations (L12–L13; Figure 6).

## 4 DISCUSSION

Species listed as Threatened or Endangered are at risk of extinction due to the adverse effects of natural and anthropogenic stressors. Climate change, either by acting alone or by exacerbating the effects of current stressors, constitutes an important new threat for many of these species (Arribas et al., 2012). Based on our results, even under a mild climate change scenario, about 25% of currently suitable snow leopard habitat in Qinghai Province is projected to be lost. Research on how snow leopards may respond to these changes will allow researchers to circumvent the challenges associated with anticipated climate changes and to tailor conservation strategies with potential species distribution shifts in mind.

Historically, snow leopards were found in all the major mountain ranges of Qinghai Province as well as in various small massifs (Schaller et al., 1988). However, due to a variety of threats to species persistence, the snow leopard's population and distribution rapidly decreased, with evidence of some local extirpation events (Li & Lü, 2014). Fortunately, the fate of snow leopards improved once they were listed as a Class I State Key Protected Wild Animal in 1989 in China (Li et al., 2016). The Chinese government has implemented conservation programs to protect and improve habitat for wildlife including the snow leopard, which serves as a recognizable flagship species for conservation. This has been very successful in some areas (Liu et al., 2016). China has recently launched an ambitious program to establish a national park system that integrates current protected areas to resolve problems associated with fragmented management (Xu et al., 2019). As the first location of the system's ten national parks, Qinghai Province has provides approximately 200,000 km^2^ of strictly protected area (Sanjiangyuan and Qilian Mountain), offering a new way for comprehensive, complete and continuous protection of at‐risk species. But whether or not this habitat can be sustained within the context of climate change remains uncertain. Therefore, the assessment of habitat vulnerability is a valuable tool to prioritize conservation strategies that reduce the impact of climate change on species such as the snow leopard.

Core distribution areas are important because they serve as connective hubs, allowing for greater genetic exchange between populations (Duan et al., 2016). Our results showed that climate change would not only shrink currently suitable snow leopard habitat along the edges of Qinghai Province, but also within their core distribution areas (e.g., Xinghai, Yushu, Maqin, and Chindu), making them more fragmented. For example, currently suitable habitat along the eastern and southern edges of Qinghai Province (e.g., Hennan and Jigzhi, which currently have a relatively small population of snow leopards based on camera‐trap data; Forestry and Environmental Protection Bureau of Hennan county, 2018; Xiao et al., 2019) were relatively isolated and fragmented, and were projected to experience the greatest degree of habitat loss. Snow leopards in these regions will be limited by natural and artificial barriers if they attempt to disperse toward core distribution areas during periods of climatic stress. Therefore, effective conservation strategies for these regions should consider the maintenance of current corridors and the potential construction of additional migration corridors to facilitate dispersal to larger core areas.

The Qinghai–Tibetan Plateau is the world's highest, largest, and youngest plateau (Erkan et al., 2011). The warming rate of the Tibetan Plateau is more than twice the Northern Hemisphere average and is predicted to continue or accelerate in this century (Peng et al., 2012; Yuke, 2019). Our results predicted that the area of suitable snow leopard habitat will decrease in future, and that mean elevations of suitable habitat will shift to higher altitudes, similar to previous findings from past studies (Li et al., 2016). The snow leopard inhabits alpine and subalpine zones that lie between the snow and tree line (Snow Leopard Network, 2014). With the temperature warming, these zones will move up toward mountaintops, resulting in an overall decrease of snow leopard habitat and increase of habitat fragmentation (Forrest et al., 2012). To make matters worse, an upward shift in the tree line will inevitably cause changes in vegetation communities, which will cause shifts in where snow leopard prey species, such as the blue sheep, forage. These areas may be closer to pastoralist communities, making snow leopards more likely to encounter and kill livestock (Aryal et al., 2013, 2014).

Protected areas provide the most effective approach for biodiversity conservation, yet they are failing to protect species from future climate change (Hannah et al., 2007). Most protected areas were established decades ago. Their locations were typically based on empirical information and expert opinion (Xu et al., 2014), without consideration for climate change (Araùjo et al., 2011). Range shifts due to climate change may cause species to move out of protected areas, thereby resulting in reducing the relevance of current fixed protected areas in future conservation strategies (Monzón et al., 2011). Our results revealed that loss of suitable snow leopard habitat would affect the conservation effectiveness of current nature reserves in Qinghai Province. These nature reserves do not adequately protect currently suitable habitat for the snow leopard (Figure 6), and they are poorly suited for accommodating species range shifts. The further construction of national parks provides an opportunity to design them while considering the impacts of climate change, such as climate induced range shifts, in China.

Assessments of vulnerability can provide valuable information about the locations that are vulnerable to climate change, enabling managers to make appropriate conservation and management strategies in light of expected future climate warming (Pacifici et al., 2015). Based on our results, conservation actions must be focused on those regions, which are predicted to suffer from large range contractions. These regions present the greatest risk to future snow leopard persistence. Vulnerability assessments also identify potential climate refugia for snow leopard within the Qinghai Province. These areas of relatively unchanged suitable habitat may facilitate species persistence during periods of climatic stress and thus warrant protection.

As a flagship species in China, snow leopards are a heavy focus for conservation efforts. The government of China has listed snow leopards in the key program of biodiversity conservation and has established two national parks (Sanjiangyuan and Qilian Mountains) specifically for protecting species in Qinghai Province (Ministry of Environmental Protection, 2011). Although the last two decades have seen remarkable advancements in our knowledge of snow leopard status and ecology, numerous gaps in knowledge remain. Sustainable conservation strategies require comprehensive information on the status and ecology of the snow leopard. The species’ response to climate change and which management strategies will be most be effective will require long‐term standardized monitoring programs. Non‐climate‐related stressors, such as domestic livestock, feral dogs, and disease, also challenge snow leopard conservation (Li et al., 2019). Domestic animals are a potential prey item for snow leopards, and they opportunistically kill livestock even if wild prey are available and abundant (Suryawanshi et al., 2017). In addition, Tibetan mastiff ownership is loose and the population is growing (Liu, 2018). Camera traps in the Qilian Mountains frequently detected Tibetan mastiffs in snow leopard distribution areas (Xue et al., 2019), indicating that predation pressure and competition could become a growing problem. Furthermore, snow leopards can harbor viruses that may negatively impact their health if the animal is experiencing environmental stress (Johansson et al., 2020). These stressors may produce synergic effects that would increase the adverse impacts of climate change on the species. Thus, mitigating these would help to minimize the net negative impact of climate change.

Validation of our results revealed that there was good concordance between known distribution of the snow leopard in Qinghai Province and the suitable habitat predicted by the model. Nevertheless, our study had some limitations. First, snow leopard habitat is impacted by other physical and biological factors. However, data on biotic and abiotic factors in Qinghai Province are rare, which restricted our choice variables for building the model. Second, our findings ignore the interactions of ecological processes on the snow leopard in response to climate change. Regardless, our results represent a reliable analysis of the impacts of climate change on snow leopard distribution and are based on the best data available for the species in Qinghai Province.

## 5 CONCLUSION

We assessed the impact of climate change on suitable snow leopard habitat, evaluated conservation effectiveness of existing nature reserves, and identified areas of climate refugia and habitat corridors in Qinghai Province, China. Under a mild climate change scenario, our results revealed that the overall area of suitable habitat was predicted to decrease in future, and that mean elevations of suitable habitat will shift upward. We suggest that future nature reserves be in areas that are currently unprotected but have climate refugia, that current migration corridors be maintained, that long‐term monitoring efforts increase, and that actions aimed at mitigating non‐climate‐related stressors continue. Our results provide valuable information for formulating strategies to mitigate the future challenges brought on by climate stress in Qinghai Province, an area which is vital to the persistence of the snow leopard in China.

## CONFLICT OF INTEREST

None declared.

## AUTHOR CONTRIBUTIONS

**Jia Li:** Conceptualization (equal); Data curation (equal); Formal analysis (equal); Investigation (equal); Methodology (equal); Resources (equal); Software (equal); Supervision (equal); Validation (equal); Writing‐original draft (equal); Writing‐review & editing (equal). **Yadong Xue:** Data curation (equal); Formal analysis (equal); Investigation (equal); Software (equal); Writing‐original draft (equal). **Charlotte E. Hacker:** Writing‐review & editing (equal). **Yu Zhang:** Data curation (equal); Investigation (equal). **Ye Li:** Investigation (equal). **Wei Cong:** Formal analysis (equal). **Lixiao Jin:** Formal analysis (equal); Investigation (equal). **Gang Li:** Investigation (equal). **Bo Wu:** Investigation (equal). **Diqiang Li:** Formal analysis (equal); Funding acquisition (equal); Investigation (equal); Software (equal); Validation (equal); Writing‐review & editing (equal). **Yuguang Zhang:** Data curation (equal); Funding acquisition (equal); Investigation (equal); Methodology (equal); Writing‐original draft (equal); Writing‐review & editing (equal).

## Supporting information

Appendix S1

## Data Availability

Climate data and MaxEnt input files for this study will be available at the Dryad after the paper publishing.

## APPENDIX 1

The code and description of climate variables, the min, max, mean, and SD values are calculated across the study area

**Table:**

Variable	Description	Unit	Type	Min	Max	Mean	SD
Bio1	Annual mean temperature	°C	Continuous	−131	129.00	−11.04	42.05
Bio2	Mean diurnal range	°C	Continuous	102	172.00	143.84	9.34
Bio3	Temperature constancy	–	Continuous	30	47.00	37.28	3.11
Bio4	Temperature seasonality	–	Continuous	5870	12,104.00	8390.32	1157.66
Bio5	Max temperature of warmest month	°C	Continuous	40	360.00	165.33	53.36
Bio6	Min temperature of coldest month	°C	Continuous	−320	−25.00	−217.37	34.81
Bio7	Annual temperature range	°C	Continuous	284	500.00	382.70	34.35
Bio8	Mean temperature of wettest quarter	°C	Continuous	−28	265.00	91.08	46.85
Bio9	Mean temperature of driest quarter	°C	Continuous	−224	116.00	−110.26	39.46
Bio10	Mean temperature of warmest quarter	°C	Continuous	−28	266.00	92.15	48.37
Bio11	Mean temperature of coldest quarter	°C	Continuous	−237	43.00	−122.64	37.24
Bio12	Annual precipitation	mm	Continuous	17	1177.00	327.26	205.11
Bio13	Precipitation of wettest month	mm	Continuous	6	231.00	75.77	40.38
Bio14	Precipitation of driest month	mm	Continuous	0	9.00	1.66	1.34
Bio15	Precipitation seasonality	–	Continuous	74	151.00	101.16	11.38
Bio16	Precipitation of wettest quarter	mm	Continuous	13	645.00	201.00	113.00
Bio17	Precipitation of driest quarter	mm	Continuous	0	35.00	6.79	5.06
Bio18	Precipitation of warmest quarter	mm	Continuous	13	645.00	200.50	112.77
Bio19	Precipitation of coldest quarter	mm	Continuous	0	35.00	7.32	5.10
Slope	Slope	°	Continuous	0	71.75	17.79	13.39
Setdes	Density of settlement	m/km^2^	Continuous	0	2039.60	69.66	150.50
Roddes	Density of road	m/km^2^	Continuous	0	103.83	7.67	8.83
Rivdes	Density of river	m/km^2^	Continuous	0	25.68	3.90	3.43
Raides	Density of Railway	m/km^2^	Continuous	0	13.75	0.57	1.54
Ele	Elevation	m	Continuous	795	6191.00	3903.61	977.34

## APPENDIX 2

Analysis of correlation coefficients to test for multicollinearity of environmental variables using Pearson correlation

**Table:**

Layer	Bio1	Bio2	Bio3	Bio4	Bio5	Bio6	Bio7	Bio8	Bio9	Bio10	Bio11	Bio12	Bio13	Bio14	Bio15	Bio16	Bio17	Bio18	Bio19	Slope	Setdes	Roddes	Rivdes	Raides	Ele
Bio1	1.00																								
Bio2	.43	1.00																							
Bio3	−.16	.30	1.00																						
Bio4	.36	.24	−.83	1.00																					
Bio5	.94	.48	−.40	.66	1.00																				
Bio6	.93	.27	.07	.06	.77	1.00																			
Bio7	.51	.47	−.69	.96	.77	.19	1.00																		
Bio8	.95	.44	−.38	.62	.99	.81	.73	1.00																	
Bio9	.91	.49	.11	.13	.79	.92	.30	.80	1.00																
Bio10	.96	.44	−.39	.62	1.00	.81	.73	1.00	.81	1.00															
Bio11	.93	.37	.18	−.01	.74	.98	.16	.78	.92	.78	1.00														
Bio12	−.06	−.26	.65	−.86	−.39	.17	−.78	−.33	.07	−.33	.26	1.00													
Bio13	−.18	−.33	.66	−.89	−.50	.07	−.84	−.44	−.05	−.44	.15	.98	1.00												
Bio14	−.01	−.27	.48	−.69	−.28	.19	−.63	−.22	.10	−.23	.26	.83	.79	1.00											
Bio15	−.41	.16	.00	.19	−.22	−.44	.10	−.27	−.30	−.27	−.49	−.51	−.40	−.61	1.00										
Bio16	−.15	−.31	.66	−.89	−.47	.10	−.83	−.41	−.01	−.41	.18	.99	1.00	.81	−.43	1.00									
Bio17	.01	−.24	.52	−.73	−.28	.21	−.65	−.23	.11	−.23	.29	.91	.85	.96	−.65	.88	1.00								
Bio18	−.16	−.31	.66	−.89	−.48	.09	−.83	−.42	−.02	−.42	.17	.99	1.00	.81	−.43	1.00	.88	1.00							
Bio19	−.03	−.26	.52	−.73	−.31	.16	−.65	−.26	.07	−.26	.24	.89	.84	.96	−.63	.86	.99	.86	1.00						
Slope	−.09	−.24	.09	−.23	−.16	.00	−.25	−.15	−.03	−.15	−.01	.25	.23	.21	−.15	.25	.23	.25	.22	1.00					
Setdes	.31	−.10	.03	−.14	.18	.37	−.09	.21	.28	.22	.38	.27	.24	.17	−.33	.24	.21	.23	.18	.13	1.00				
Roddes	.40	−.12	−.05	−.09	.26	.44	−.04	.30	.32	.31	.45	.28	.24	.21	−.42	.24	.24	.24	.21	.12	.82	1.00			
Rivdes	.00	−.13	.36	−.48	−.19	.12	−.42	−.16	.05	−.16	.18	.56	.55	.50	−.38	.56	.52	.56	.50	.16	.33	.40	1.00		
Raides	.28	−.06	−.30	.26	.31	.23	.24	.32	.16	.32	.20	−.18	−.19	−.15	−.15	−.19	−.16	−.20	−.18	−.05	.41	.50	−.01	1.00	
Ele	−.89	−.32	.52	−.67	−.95	−.71	−.76	−.95	−.71	−.96	−.68	.33	.45	.27	.32	.42	.24	.43	.28	.09	−.25	−.34	.16	−0.34	1.00
