# Supplementary material for: Projected impacts of climate change on snow leopard habitat in Qinghai Province, China
Source: Ecol Evol. 2021 Nov 18;11(23):17202–18. doi: 10.1002/ece3.8358 (PMC8668752; doi:10.1002/ece3.8358)
Supplement: Supplementary file 1 — Appendix S1 [file ECE3-11-17202-s001.docx]

**APPENDIX S1** Data sources for records of snow leopard in Qinghai Province, China.

| City or Prefecture | County | Numbers of records | Data source code |
| --- | --- | --- | --- |
| Xining city | Datong | 1 | [1/21] |
| Haidong city | Huzhu | 2 | [2/21] |
| Haibei Tibetan  Autonomous  Prefecture | Menyuan | 10 | [2-3] |
|  | Qilian | 62 | [2-3/6] |
|  | Gangca | 6 | [2-3/21] |
| Hainan Tibetan  Autonomous  Prefecture | Tongren | 1 | [21] |
|  | Henan | 32 | [2/4-5] |
|  | Gonghe | 1 | [21] |
|  | Guide | 1 | [21] |
|  | Xinghai | 12 | [2/5/7/12/21] |
|  | Guinan | 1 | [21] |
| Guoluo Tibetan  Autonomous  Prefecture | Maqin | 73 | [2/5/8/13] |
|  | Baima | 1 | [2] |
|  | Gade | 1 | [21] |
|  | Dari | 1 | [2] |
|  | Jigzhi | 26 | [2/5] |
|  | Maduo | 20 | [2/5/9] |
| Yushu Tibetan  Autonomous  Prefecture | Yushu | 129 | [2/5-6/8/11/15-17] |
|  | Zadoi | 296 | [2/5/10/16-17] |
|  | Chindu | 10 | [2/8] |
|  | Zhiduo | 344 | [2/5-6/8-11/13/15/17-20] |
|  | Nangqian | 91 | [2/5/8-10/17-18] |
|  | Qumarleb | 40 | [2/8/10/17/19] |
| Haixi Mongolian  and Tibetan  Autonomous  Prefecture | Haixi | 5 | [2] |
|  | Golmud | 21 | [2/5/21] |
|  | Delingha | 12 | [2-3] |
|  | Wulan | 2 | [2/21] |
|  | Dulan | 63 | [2/14-15/19-20] |
|  | Tianjun | 27 | [2-3] |

[1] Ma GQ, Liang BK, Ma YZ, Zhang YJ. 2009. Qinhai Datong Beichuan Heyuanqu nature reserve biodiversity. Beijing: Beijing Press.

[2] China snow leopard protection network. 2018. Study of snow leopard survey and conservation, China (2018). <http://www.snowleopardchina.org/>.

[3] Infrared cameras and field investigation in Qilian national Park, 2017-2020.

[4] Forestry and Environmental Protection Bureau of Henan County. 2018. Camera-trapping survey of the snow leopard and other wildlife in Hennan country of Huangnan prefecture, Qinghai Province (2014-2016).

[5] Xiao L Y, Chen C, Wan W H, Zhang D H, Wang Y C, Tsedan, Hou P, Li J, Yang X, L Z, Liu Y P. 2019. Defining conservation priority areas of snow leopard habitat in the Sanjiangyuan Region. Biodiversity Science, 27(9): 943-950.

[6] Zhang Y G, Hacker C, Zhang Y, Xue Y D, Wu L J, Dai Y C, Luo P, Xieran N M, Janecka J E, Li D Q. 2019. An analysis of genetic structure of snow leopard populations in Sanjiangyuan and Qilianshan Naitonal Parks. Acta Theriologica Sinica, 39(4): 442-449.

[7] Li J, Liu F, Zhang Y, Li G L, Li D Q. 2016. Using camera traps to survey mammals in Zhongtie-Jungong area of Sanjiangyuan national nature reserve, Qinghai province. Biodiversity Science, 24(6): 709-713.

[8] Cai X W, Jiang F, Gao H M, Qin W, Wu T, Zhang J J, Cai Z Y, Zhang T Z. 2019. Habitat suitability analysis of snow leopard (*Panthera uncia*) and bharal (*Pseudois nayaur*) in the Sanjiangyuan National park. Acta Theriologica Sinica, 39(4): 397-409.

[9] National Development and Reform Commission. 2018. Master plan of Sanjiangyuan National park (2017-2025).

[10] Special plan for ecological protection of Sanjiangyuan National Park (2017-2035). 2018. Sanjiangyuan National Park Administration, Chinese Academy of Environmental Science.

[11] Infrared cameras and field investigation in Sanjianyuan national Park, 2015-2018.

[12] Field investigation in Maqin and Xinghai county, 2015.

[13] Qinghai Hoh Xil National Nature Reserve Administration. 2018. Qinghai Kekexili national nature reserve master plan (2018-2027).

[14] Infrared cameras and field investigation in Dulan county, 2018-2020.

[15] Alexander J S, Zhang C C, Shi K, Rirdan P. 2016. A spotlight on snow leopard conservation in China. Integrative Zoology, 11(4): 308-321.

[16] Zheng J. 2011. The study of Qinghai nature reserve. Xining: Qinghai People's Publishing House.

[17] Zhou Y Y, Feng J Z, Duo H R, Yang H L, Li J, Li D Q, Zhang Y G. 2014. Population survey and genetic diversity of snow leopard (*Uncia uncia*) in Qnghai-Tibet Plateau as revealed by fecal DNA. Acta Theriologica Sinica, 34(2): 138-148.

[18] Zhou Y Y, Duo H R, Xue Y D, Li D Q, Feng J Z, Zhang Y G. 2015. Genetic diversity analysis of microsatellite DNA in snow leopard (*Panthera uncia*). Chinese Journal of Zoology, 50(2): 161-168.

[19] Zhang Y G, Janecka J E, Li D Q, Duo H R, Jackson R, Murphy W J. 2008. Population survey and genetic diversity of snow leopards *Panthera uncia* as revealed by fecal DNA. Acta Zoologica Sinica, 54(5): 762-766.

[20] Zhang Y G, He L, Duo H R, Li D Q, Jin K. 2009. A preliminary study on the population genetic structure of snow leopard (*Unica unica*) in Qinghai Province utilizing fecal DNA. Acta Zoologica Sinica, 9(3): 310-315.

[21] Others such as personal communication, new reports and field observation.
